# Serial Coronary Plaque Composition After Inclisiran Initiation Assessed by Automated CCTA: A Retrospective Pilot Study

**DOI:** 10.3390/diagnostics16183009

**Published:** 2026-09-17

**Authors:** Kentaro Kawada, Masafumi Suzuki, Junji Mochizuki, Hiroaki Matsumi, Daiji Miura, Yoshiki Hata

**Affiliations:** 1 Department of Cardiology, Minamino Cardiovascular Hospital, 1-25-1 Hyoue, Hachioji, Tokyo 192-0918, Japan; 2 Department of Radiology, Minamino Cardiovascular Hospital, 1-25-1 Hyoue, Hachioji, Tokyo 192-0918, Japan; 3 Division of Basic and Clinical Medicine, Nagano College of Nursing, 1694 Akaho, Komagane, Nagano 399-4117, Japan; miura-daiji@nagano-nurs.ac.jp

**Keywords:** inclisiran, coronary computed tomography angiography, CCTA, artificial intelligence, coronary plaque, low-density non-calcified plaque, plaque remodeling, LDL cholesterol

## Abstract

**Background/Objectives**: Inclisiran provides durable low-density lipoprotein cholesterol (LDL-C) lowering, but serial coronary plaque composition after treatment initiation remains incompletely characterized. This study evaluated temporal plaque changes using automated coronary computed tomography angiography (CCTA). **Methods**: In this uncontrolled, retrospective, observational pilot study, consecutive patients who initiated inclisiran between 1 January 2024 and 29 April 2026 were screened. A random-intercept mixed-effects model included every eligible quantitative plaque observation obtained by the prespecified data cutoff. **Results**: Of the 230 screened patients, 215 contributed 468 plaque observations (baseline, *n* = 186; 3 months, *n* = 196; 15 months, *n* = 86); 74 had all three examinations. The LDL-C levels decreased from 117.4 mg/dL (95% confidence interval [CI], 113.3–121.6) to 45.9 mg/dL (39.5–52.3) at 15 months. No significant time effect was detected for the total plaque volume (*p* = 0.713). Low-density non-calcified plaque (LDNC) decreased from 27.3 mm^3^ (23.1–31.6) to 22.7 mm^3^ (18.0–27.4; overall *p* = 0.0035), whereas calcified plaque increased from 56.5 mm^3^ (45.6–67.3) to 67.1 mm^3^ (55.9–78.2; *p* < 0.0001). The adjusted baseline-to-15-month differences were −4.64 mm^3^ for LDNC (95% CI, −7.54 to −1.74; Bonferroni-adjusted *p* = 0.0052) and +10.60 mm^3^ for calcified plaque (6.60–14.60; adjusted *p* < 0.0001). In the tube voltage-adjusted sensitivity analysis of 107 patients and 278 observations, these findings were maintained. **Conclusions**: Patients initiating inclisiran showed temporal changes in attenuation-defined plaque composition. The uncontrolled design, incomplete acquisition data in the expanded cohort, and technical variability preclude causal or histologic interpretation.

## 1. Introduction

Inclisiran is a small interfering RNA that inhibits the hepatic synthesis of proprotein convertase subtilisin/kexin type 9 (PCSK9), thereby increasing low-density lipoprotein receptor recycling and lowering circulating low-density lipoprotein cholesterol (LDL-C). In the pivotal ORION-10 and ORION-11 trials, inclisiran treatment administered on days 1 and 90 and every 6 months thereafter produced approximately 50% placebo-corrected LDL-C reductions over 18 months, and subsequent pooled and extension analyses supported sustained lipid lowering with an infrequent maintenance dosing strategy [1,2,3]. Although the LDL-C-lowering efficacy of inclisiran is well established, longitudinal changes in coronary plaque burden and composition after its initiation remain incompletely characterized, particularly in routine clinical settings.

The biological rationale for evaluating the coronary plaque response after PCSK9-targeted therapy is supported by prior imaging studies on monoclonal antibody-type PCSK9 inhibition. In the GLAGOV randomized trial, evolocumab added to statin therapy achieved substantially lower LDL-C levels and greater intravascular ultrasound-defined coronary atheroma regression than the placebo [4]. More recent multimodal intracoronary imaging studies, including HUYGENS and PACMAN-AMI, further suggested that intensive PCSK9 inhibition in addition to statin therapy can favorably modify the plaque phenotype, including fibrous-cap thickening, lipid-arc reduction, macrophage signal reduction, and plaque regression [5,6]. These studies provide an important mechanistic context, but their findings cannot be directly extrapolated to inclisiran or noninvasive CCTA-based plaque characterization.

Serial coronary computed tomography angiography (CCTA) has emerged as a practical and noninvasive approach for assessing the progression of coronary atherosclerosis and treatment-associated plaque remodeling over time [7,8]. In serial CCTA cohorts, statin therapy has been associated not only with slower overall plaque progression but also with compositional transformation, including reduced low-attenuation or non-calcified plaque components and increased calcified or higher-density components [9,10]. Therefore, CCTA-based plaque composition provides a clinically interpretable framework for evaluating whether profound LDL-C lowering is accompanied by a shift toward a more stable plaque phenotype, even when the total plaque volume changes are modest.

Simultaneously, quantitative CCTA plaque assessment remains methodologically complex. Contemporary consensus documents emphasize the need for standardized acquisition, segmentation, reporting, and interpretation of plaque burden and composition, while also recognizing that attenuation-based thresholds and software-specific definitions may differ across platforms [11,12,13]. Artificial intelligence (AI)-assisted CCTA tools may improve reproducibility, reduce operator dependence, and facilitate scalable longitudinal plaque monitoring. However, real-world evidence using fully automated, nonmanual correction workflows remains limited [14,15].

Accordingly, we aimed to evaluate longitudinal changes in coronary plaque composition after the initiation of inclisiran treatment using fully automated AI-assisted CCTA. We specifically assessed changes in the total plaque volume, total non-calcified plaque (NCP), low-density non-calcified plaque (LDNC), and calcified plaque volume; evaluated the relationship between LDL-C reduction and LDNC reduction; and performed hypothesis-generating subgroup analyses according to acute coronary syndrome (ACS) versus chronic coronary syndrome (CCS) presentation.

## 2. Materials and Methods

### 2.1. Study Design and Patient Population

This single-center, retrospective, observational pilot study was conducted at Minamino Cardiovascular Hospital. Follow-up CCTA at approximately 3 and 15 months was prospectively scheduled as part of an institutional clinical-care protocol for assessing treatment response after inclisiran initiation and was not performed specifically for the present research. The present study retrospectively analyzed existing imaging and clinical data generated during routine care. Consecutive patients who initiated inclisiran between 1 January 2024 and 29 April 2026 were screened.

The final data lock date was 29 April 2026. Because the treatment-initiation window extended through the data lock date, patients who initiated treatment later in the window had not yet accrued 15 months of follow-up at data lock. Baseline CCTA was defined as an eligible routine care examination performed before or on the day of inclisiran initiation. No maximum look-back interval was imposed in the primary retrospective extraction; examinations performed more than 90 days before treatment were retained because they met the prespecified pretreatment baseline definition, and their influence was assessed in the sensitivity analysis. The 3- and 15-month examinations were identified using prespecified clinical windows of ±14 days. Examinations obtained after the data lock date were not included. The primary longitudinal analysis included every eligible quantitative plaque measurement available by the data lock date, irrespective of whether the patient completed 15 months of imaging.

### 2.2. CCTA Acquisition Protocol

The CCTA examinations were performed using an APEX Elite 256-row scanner (GE Healthcare, Chicago, IL, USA) with non-helical, prospectively electrocardiogram-triggered acquisition, automatic exposure control, and 0.625-mm slice thickness. The images were reconstructed using the HD Standard kernel and a strong level of iterative reconstruction. The cardiac phase with the least motion artifact was selected, and SnapShot Freeze 2.0 motion-correction processing was applied. Tube voltage was 120 or 140 kVp according to the patient and scanning conditions. Iodinated contrast medium (240 mg I/mL) was administered over a fixed 13-s injection period, followed by a 30-mL saline flush at the same injection rate. To reduce differences in intraluminal coronary attenuation between tube voltage protocols, the iodine delivery rate was adjusted to 20 mg I/kg/s at 120 kVp and 23 mg I/kg/s at 140 kVp. No post-processing normalization of plaque attenuation according to tube voltage was performed. Sublingual nitroglycerin was administered unless clinically contraindicated; beta-blockers were not administered routinely. CTDIvol and dose-length product (DLP) were retrospectively retrieved when available. The effective dose was estimated as DLP multiplied by 0.014 mSv/(mGy·cm) [16].

### 2.3. AI-Assisted Plaque Quantification and Outcomes

The coronary plaque analysis was performed using CoronaryDoc (version 2.0.0.001; Shukun Technology, Beijing, China). Quantification was fully automated without manual correction. The same software version was used for all examinations. All automated outputs underwent visual quality control review. No examination was excluded because of an unanalyzable output or complete segmentation failure. The analyst was not blinded to the examination time point or clinical information. According to the software manual, coronary arteries with a reference vessel diameter of ≥1.5 mm were intended for automated analysis; however, smaller distal vessels could also be recognized depending on attenuation and the anatomical course. No additional post-processing exclusion was applied solely on the basis of the vessel diameter. The same coronary arteries were evaluated at each available time point, while the analyzed range was automatically extracted independently by the software at each examination. Strict point-by-point anatomical coregistration of identical coronary segments was not performed. Previously implanted stents were automatically identified and excluded. Patients undergoing percutaneous coronary intervention during the follow-up remained eligible, but only the treated coronary segments, including newly stented segments, were excluded; untreated segments remained available for the longitudinal analysis. Plaque components were prespecified as total NCP (−30 to <350 HU), investigator-defined LDNC (−30 to <75 HU, a subset of NCP), and calcified plaque (≥350 HU). The 75-HU upper threshold was selected on the basis of histopathologic validation showing quantitative agreement between the CCTA-derived low-attenuation plaque area and histologic lipid-rich plaque area [17]. These thresholds were applied uniformly to all patients and time points, and component volumes were directly quantified by the software. The primary outcomes were longitudinal changes in the LDL-C, total plaque, total NCP, LDNC, and calcified plaque volumes.

### 2.4. Statistical Analysis

Baseline characteristics are presented as mean ± standard deviation for continuous variables and as number (percentage) for categorical variables. Longitudinal model-derived values are presented as estimated marginal means with 95% confidence intervals (CIs).

Longitudinal changes were assessed using a linear mixed-effects model with time modeled categorically as a fixed effect and patient as a random intercept, yielding a compound-symmetry covariance structure. All the eligible observations available by the data lock date were included without imputation. Model-estimated means, 95% CIs, and overall time effects are reported. Three pairwise time contrasts were evaluated within each outcome with Bonferroni-adjusted *p*-values. To address multiplicity across the five overall outcome tests, Benjamini–Hochberg false discovery rate-adjustment was additionally considered. As an acquisition-parameter sensitivity analysis, the tube voltage (120 versus 140 kVp) was included as a time-varying fixed-effect covariate in the subset with linkable tube voltage data. Plaque volume residuals were right-skewed; therefore, complete-case and prespecified sensitivity analyses were used to assess robustness. Results were interpreted as exploratory rather than confirmatory.

The paired baseline-to-15-month LDL-C/LDNC subset (*n* = 64) was analyzed using the Pearson and Spearman correlations. Pearson 95% CIs were calculated using the Fisher z transformation. The sensitivity analysis excluded observations with an absolute standardized value > 2 for either change score. The ACS-versus-CCS analyses included time-by-group interaction terms. All tests were two-sided; *p* < 0.05 was considered statistically significant, with adjusted *p*-values reported where applicable.

### 2.5. Use of Generative AI in Manuscript Preparation

Generative AI was used only to assist manuscript preparation and revision. ChatGPT, including the Codex workspace (OpenAI, San Francisco, CA, USA; web-based service accessed on 5 September 2026; the specific model version was not displayed), was used for statistical code development, internal consistency checking, language refinement, and document formatting. Manus AI (Manus; https://manus.im; web-based service accessed on 5 September 2026; no version number was displayed) was used only at the final revision stage to check language and consistency and suggest editorial revisions. Neither tool was used to define the study design, generate or alter primary study data, or quantify CCTA outcomes; CCTA plaque quantification was performed separately with CoronaryDoc as described earlier. All AI-assisted code, analytical output, figures, and text were reviewed and verified by the authors. The study statistician independently reproduced the principal mixed-effects analyses, and the authors take full responsibility for the content of this publication.

### 2.6. Ethics Statements

This study was conducted in accordance with the Declaration of Helsinki, and approved by the Institutional Review Board of Minamino Cardiovascular Hospital (approval number: MJ-108; approval date: 27 April 2026). Written informed consent for participation and for the use of clinical data for research purposes was obtained from all participants.

## 3. Results

### 3.1. Patient Flow and Baseline Characteristics

**Figure 1:**
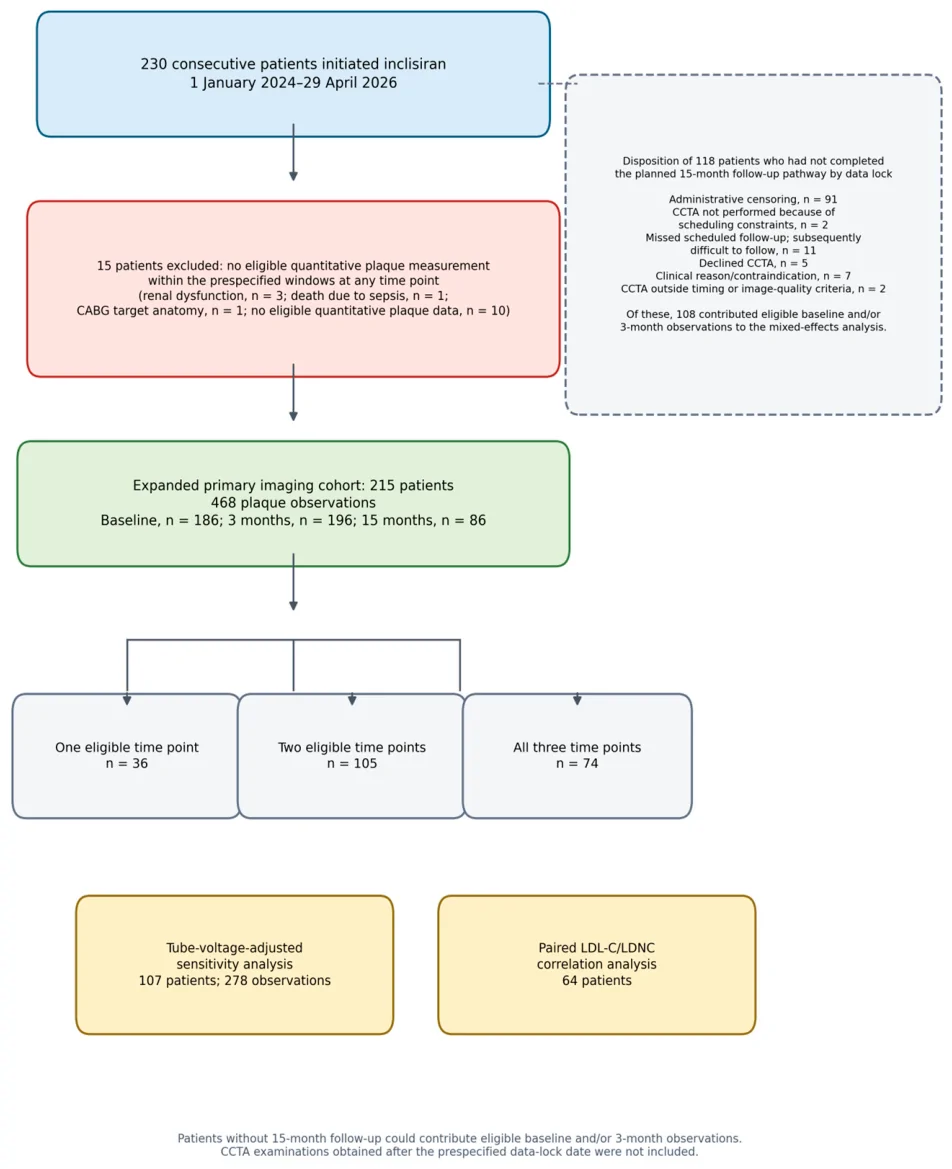
Study flow diagram. Of the 230 consecutive patients initiating inclisiran, 15 had no eligible quantitative plaque measurement at any prespecified time point, leaving 215 patients in the expanded primary imaging cohort. Among the 118 patients who had not completed the planned 15-month follow-up pathway by the data lock date, 91 were administratively censored before reaching 15 months, 2 reached 15 months but CCTA could not be performed because of scheduling constraints, 11 did not attend the scheduled follow-up and were subsequently difficult to follow, 5 declined CCTA, 7 had a clinical reason or contraindication, and 2 had CCTA outside the timing or image-quality criteria. Of these 118 patients, 108 contributed eligible baseline and/or 3-month observations to the mixed-effects analysis. The expanded cohort contributed 468 plaque observations: 186 at baseline, 196 at 3 months, and 86 at 15 months. Thirty-six patients contributed one time point, 105 contributed two, and 74 contributed all three. Examinations obtained after 29 April 2026 were excluded. The tube voltage-adjusted sensitivity analysis included 107 patients and 278 observations; the paired LDL-C/LDNC correlation subset included 64 patients. CCTA, coronary computed tomography angiography; LDL-C, low-density lipoprotein cholesterol; LDNC, low-density non-calcified plaque.

Of the 230 patients screened, 215 had at least one eligible quantitative plaque measurement by the data lock date and comprised the expanded primary imaging cohort (Figure 1). The baseline characteristics of this cohort are summarized in Table 1. Sixteen 15-month examinations obtained after the data lock date were excluded. Sixty-four patients had paired baseline and 15-month LDL-C and LDNC measurements for the correlation analysis. In the 74 complete cases, the median interval from baseline CCTA to first inclisiran administration was 2 days (interquartile range, 1–6.75 days; range, 0–393 days); three baseline examinations preceded treatment by more than 90 days. These remote examinations were retained because they met the prespecified pretreatment baseline definition. In the sensitivity analysis restricted to 71 patients with baseline CCTA within 90 days, no significant time effect was detected for the total plaque volume (overall *p* = 0.722), whereas LDNC decreased (*p* = 0.0042), total NCP decreased (*p* = 0.0451), and calcified plaque increased (*p* < 0.0001).

The tube voltage data were linkable to 278 eligible plaque observations in 107 patients. Among the 97 patients with two or more such examinations, 45 (46.4%) had the same tube voltage at all the available time points and 52 (53.6%) had at least one change. Among the 74 patients with all three examinations, 31 (41.9%) had the same tube voltage throughout.

### 3.2. Longitudinal Changes in the LDL-C Levels and Plaque Volumes

**Figure 2:**
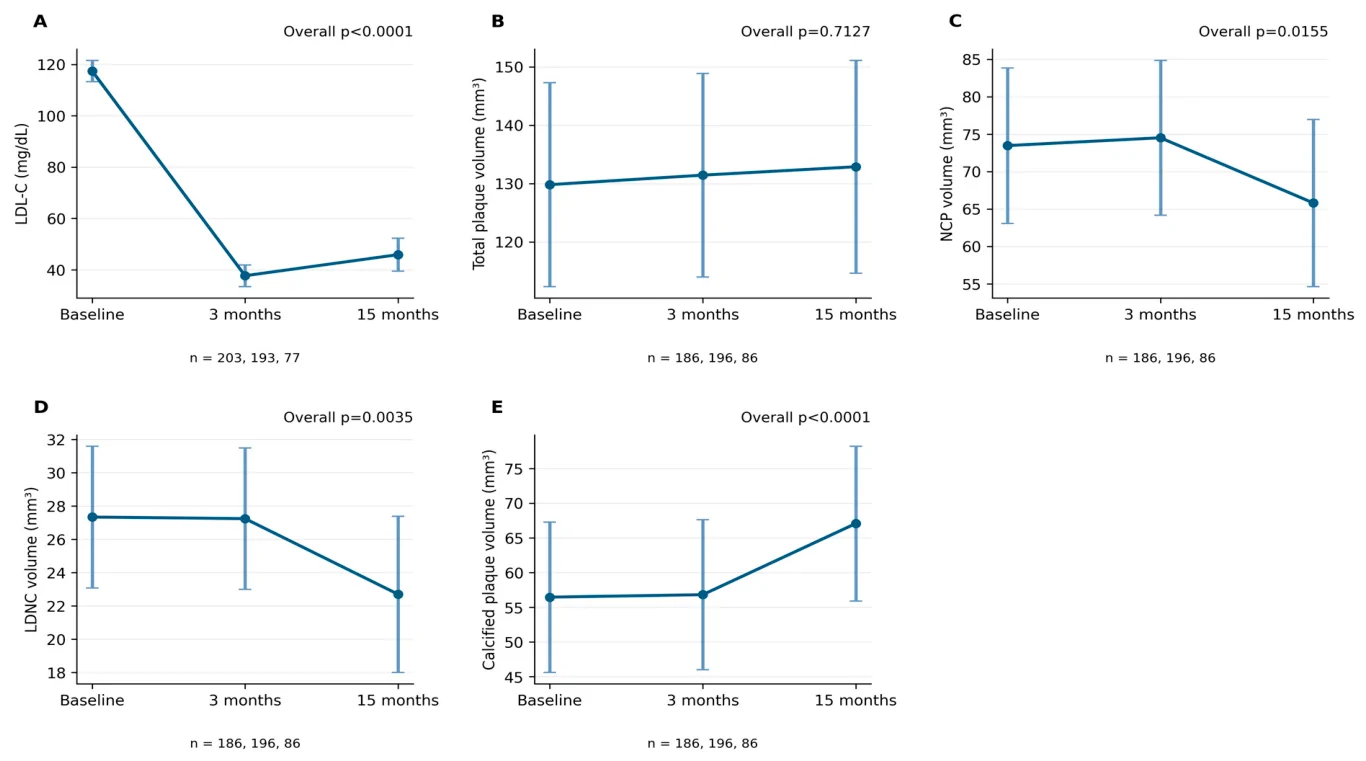
Model-estimated longitudinal changes with 95% CIs and numbers of observations at each time point in the expanded cohort: (**A**) LDL-C, (**B**) total plaque volume, (**C**) NCP, (**D**) LDNC, and (**E**) calcified plaque volume. NCP, non-calcified plaque; LDNC, low-density non-calcified plaque; CI, confidence interval.

The numbers of observations contributing to the mixed-effects models are shown in Table 2 and Figure 2A–E. The LDL-C levels decreased from an estimated 117.4 mg/dL at baseline to 37.7 mg/dL at 3 months and 45.9 mg/dL at 15 months (overall *p* < 0.0001).

Radiation dose data were available for 88 examinations at baseline, 98 at 3 months, and 87 at 15 months. The mean CTDIvol values were 21.6 ± 12.3, 20.2 ± 10.5, and 16.7 ± 9.1 mGy, respectively; corresponding DLP values were 336.0 ± 199.0, 304.6 ± 171.2, and 250.7 ± 136.5 mGy·cm. The estimated effective doses were 4.70 ± 2.79, 4.26 ± 2.40, and 3.51 ± 1.91 mSv, respectively. The mean heart rates were 69.7 ± 16.4 bpm (*n* = 87), 65.9 ± 12.4 bpm (*n* = 97), and 63.4 ± 11.0 bpm (*n* = 86), respectively (Table S1).

**Figure 3:**
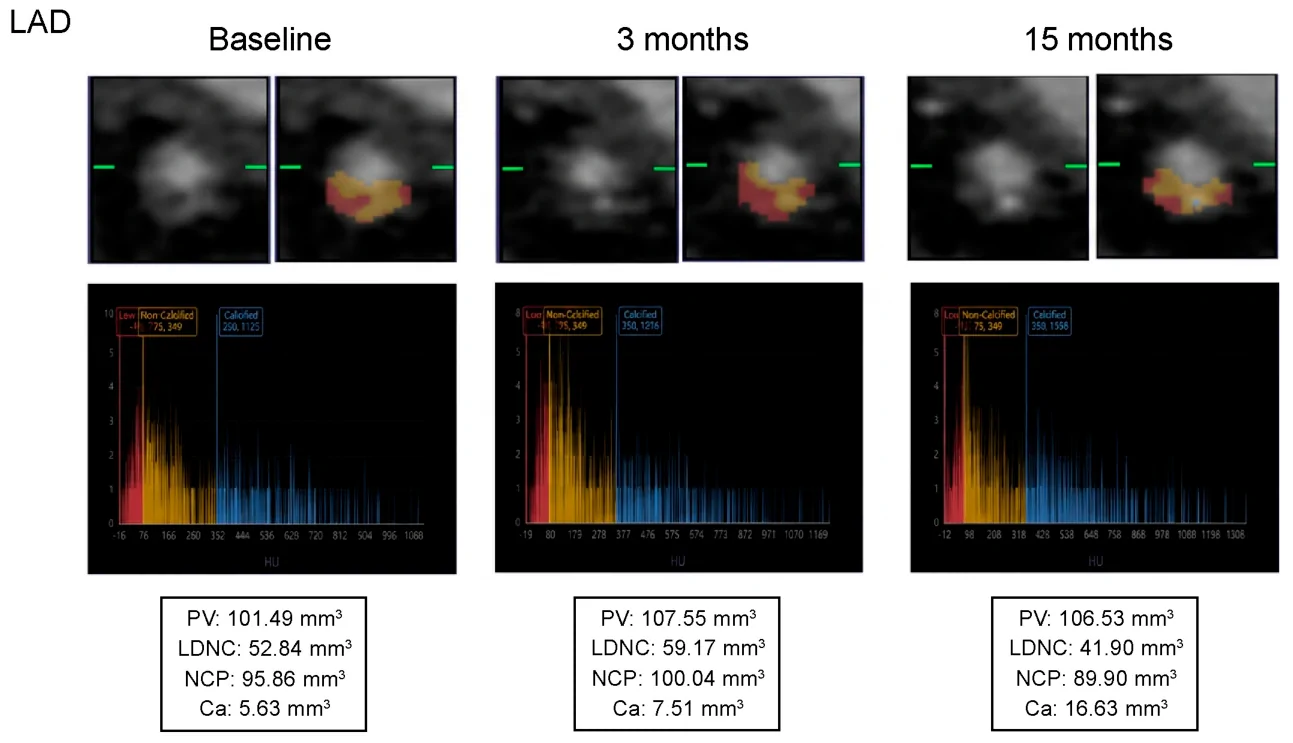
Illustrative serial AI-assisted CCTA analysis of the coronary plaque composition. The original coronary short-axis images and attenuation histograms are shown without modification. Panels depict gray-scale images and color overlays, attenuation histograms in HU, and quantitative plaque metrics. In the color overlays and attenuation histograms, red indicates LDNC (<75 HU), yellow indicates the remaining non-calcified plaque component (75–349 HU), and blue indicates calcified plaque (≥350 HU). Respective exact values for Case 1 across baseline, 3 months, and 15 months are as follows: total plaque volume, 101.49, 107.55, and 106.53 mm^3^; total non-calcified plaque, 95.86, 100.04, and 89.90 mm^3^; LDNC, 52.84, 59.17, and 41.90 mm^3^; and calcified plaque, 5.63, 7.51, and 16.63 mm^3^. This case is strictly illustrative and does not represent average cohort changes. AI, artificial intelligence; CCTA, coronary computed tomography angiography; HU, Hounsfield units; LDNC, low-density non-calcified plaque; NCP, non-calcified plaque.

No significant time effect was detected for the total plaque volume (overall *p* = 0.713). Total NCP showed an overall time effect (*p* = 0.0155)—its baseline-to-15-month difference was −7.67 mm^3^ (95% CI, −13.79 to −1.54; Bonferroni-adjusted *p* = 0.0424). LDNC decreased from baseline to 15 months by −4.64 mm^3^ (95% CI, −7.54 to −1.74; adjusted *p* = 0.0052). Calcified plaque increased by +10.60 mm^3^ (95% CI, 6.60–14.60; adjusted *p* < 0.0001). The complete-case analysis supported the LDNC and calcified plaque findings (Figure 3). In the tube voltage-adjusted sensitivity analysis of 107 patients and 278 observations, the baseline-to-15-month differences remained significant for LDNC (−4.02 mm^3^, 95% CI, −7.30 to −0.75; adjusted *p* = 0.0480) and calcified plaque (+10.27 mm^3^, 95% CI, 6.16–14.38; adjusted *p* < 0.0001), whereas total NCP was not significant.

Results of the sensitivity analyses supported the principal compositional findings. In the 74 complete cases, no significant time effect was detected for the total plaque volume (*p* = 0.594); the baseline-to-15-month differences were −5.67 mm^3^ for LDNC (Bonferroni-adjusted *p* = 0.0041) and +12.39 mm^3^ for calcified plaque (adjusted *p* < 0.0001), whereas total NCP did not remain significant after pairwise correction. The analyses of the previously specified timing and no medication change in the original complete-case cohort also supported the direction of LDNC reduction and calcified plaque increase. The tube voltage-adjusted results are reported in Table S3.

### 3.3. Correlation Between LDL-C Reduction and LDNC Volume Reduction

**Figure 4:**
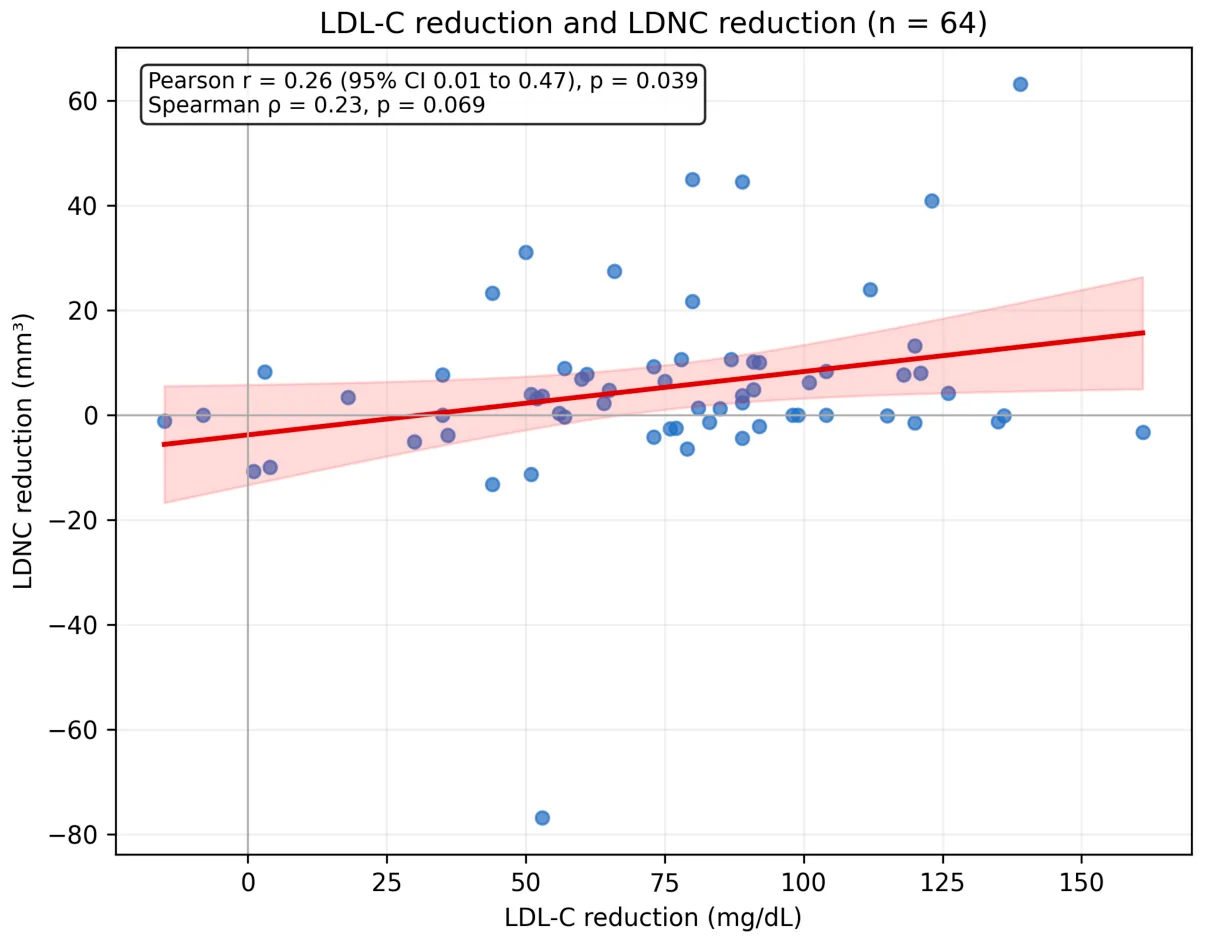
Correlation between baseline-to-15-month LDL-C reduction and LDNC reduction (*n* = 64). Blue dots represent individual observations, the red line represents the fitted linear regression line, and the pink shading represents the 95% confidence band. Pearson r = 0.26 (95% CI: 0.01–0.47; *p* = 0.039); Spearman rho = 0.23 (*p* = 0.069). The association was not significant after exclusion of the prespecified standardized-value outliers. CI, confidence interval; LDNC, low-density noncalcified plaque; LDL-C, low-density lipoprotein cholesterol.

In the paired subset (*n* = 64), LDL-C reduction correlated weakly with LDNC reduction according to the Pearson analysis, whereas the Spearman correlation was not statistically significant (Figure 4, Table S4). Eight observations met the prespecified standardized-value criterion; after their exclusion, neither the Pearson (r = 0.15; *p* = 0.280) nor the Spearman correlation (rho = 0.14; *p* = 0.291) was significant. Therefore, the correlation finding was considered non-robust and exploratory.

### 3.4. Exploratory ACS-Versus-CCS Subgroup Analysis

The exploratory ACS-versus-CCS analyses in the expanded cohort showed no significant time-by-group interaction for total plaque (*p* = 0.954), NCP (*p* = 0.748), LDNC (*p* = 0.439), or calcified plaque (*p* = 0.846) (Table S5). The LDL-C trajectory differed between the groups (interaction *p* = 0.0008); these subgroup analyses remained exploratory.

## 4. Discussion

In this single-center, retrospective pilot study, automated CCTA showed temporal changes in attenuation-defined coronary plaque composition after inclisiran initiation. Incorporating every eligible quantitative plaque observation available by the prespecified data cutoff date increased the primary cohort from 107 to 215 patients. The LDL-C levels decreased markedly; no significant time effect was detected for the total plaque volume; LDNC and total NCP decreased; and calcified plaque increased. The principal detectable imaging signal over 15 months was, therefore, a measured compositional shift rather than global plaque regression. These findings do not establish biological plaque stabilization or an inclisiran effect.

This observation should be interpreted within the broader literature on intensive lipid lowering and plaque stabilization. Invasive imaging trials of monoclonal antibody-type PCSK9 inhibition, such as GLAGOV, HUYGENS, and PACMAN-AMI, have shown that very low LDL-C levels achieved with statin therapy can be accompanied by coronary atheroma regression and favorable plaque phenotype changes [4,5,6]. Our study differs fundamentally from these trials because it evaluated inclisiran, used CCTA rather than intravascular ultrasound/optical coherence tomography, lacked a randomized control group, and was conducted in routine practice. Nevertheless, the direction of the observed changes is biologically plausible and concordant with the concept that intensive LDL-C reduction promotes plaque stabilization.

The combination of no significant time effect in total plaque volume with compositional change is consistent with results of the serial CCTA studies of intensive lipid lowering, in which reductions in lower-attenuation or non-calcified components may accompany increased calcification or plaque density [9,10]. LDNC was an investigator-defined quantitative component (−30 to <75 HU), not a direct histologic measurement. Importantly, LDNC reduction and calcified plaque increase remained significant after adjustment for tube voltage as a time-varying covariate in the 107-patient acquisition data subset. Nevertheless, attenuation measurements remain influenced by the tube voltage, luminal enhancement, reconstruction, partial-volume effects, and analysis platform. Therefore, the observed pattern should be interpreted as a software-specific attenuation-defined compositional change rather than as evidence of histologic transformation, biological stabilization, or causality.

Most patients received background statin therapy. Among the 74 complete cases, 65 had no recorded change in statin or ezetimibe therapy during the follow-up. In this no-change sensitivity cohort, the LDNC reduction and calcified plaque increase were preserved while the total plaque volume showed no significant time effect; the overall change in total NCP was not statistically significant. These findings reduce but do not eliminate concern that the principal compositional results were driven solely by changes in concomitant lipid-lowering therapy. Residual confounding by treatment intensity, adherence, and other risk factor management remains possible.

The fully automated AI workflow is another important methodological aspect of the current study. Manual correction can improve individual segmentation accuracy in selected cases; however, it also introduces operator dependence and limits scalability. By avoiding manual correction, the present analysis approximated how AI-assisted plaque quantification could function in routine clinical workflows and minimized investigator subjectivity. Simultaneously, this design choice may have allowed for residual segmentation, vessel-boundary, or component-classification errors. Therefore, the results should be regarded as workflow-level, software-specific observations rather than definitive histopathological characterization of plaque composition.

The Pearson association between the LDL-C and LDNC reductions was weak and was not corroborated by the findings of the Spearman analysis or outlier sensitivity analysis. Accordingly, this result should not be considered robust evidence of a dose–response relationship. Change-score correlations are also susceptible to baseline values, regression to the mean, and measurement error. Thus, the correlation analysis provides, at most, exploratory support for a possible relationship and does not materially strengthen causal interpretation.

The exploratory ACS-versus-CCS subgroup analyses did not identify statistically significant time-by-group interactions for any plaque metric; however, LDL-C trajectories differed between the groups. The study was not powered for interaction testing, and patients with ACS and those with CCS may differ in plaque biology, systemic inflammation, treatment intensity, and follow-up indications. Therefore, these subgroup findings should be viewed as descriptive reference data rather than as evidence of an equivalent treatment response.

This study has some limitations. Its retrospective single-center design and absence of an untreated or active-comparator group preclude causal attribution to inclisiran. Although the expanded mixed-effects analysis included every eligible quantitative plaque observation available by the prespecified data cutoff date, only 74 patients had complete three-time-point imaging, 36 contributed a single time point, and the missing data mechanism was not empirically verified. The 15-month observations necessarily came from patients who initiated treatment earlier, creating administrative censoring and potential selection bias. Although the key compositional findings persisted after excluding the remote baseline examinations, such scans may not precisely represent plaque status immediately before treatment initiation. The tube voltage data for the additionally incorporated baseline and 3-month examinations were not systematically available; therefore, tube voltage adjustment was restricted to 107 patients, and residual acquisition-related confounding in the expanded cohort cannot be excluded. In that subset, LDNC reduction and calcified plaque increase were maintained after adjustment, but the LDNC result was borderline. Luminal enhancement was not included in the model, and residual effects of contrast attenuation, reconstruction, partial-volume effects, and software-specific thresholds remain possible. Visual quality control was performed by an analyst who was not blinded, although no manual correction was made. Strict point-by-point coronary coregistration was not performed, and minor differences in analyzed boundaries remain possible. Concomitant treatment, ACS-related healing, intervention, adherence, and other risk factor management may also have contributed. Finally, this imaging biomarker study was not powered for clinical outcomes or subgroup interactions and did not assess repeatability. Despite these limitations, this study provides hypothesis-generating data on automated serial plaque assessment in routine clinical practice.

## 5. Conclusions

Among the selected patients who initiated inclisiran, automated CCTA showed temporal reductions in investigator-defined LDNC and increases in the calcified plaque, while no significant time effect was detected for the total plaque volume. These attenuation-defined changes were observed without a comparator group and cannot establish an inclisiran effect, histologic transformation, plaque stabilization, clinical utility, or reproducibility. Thus, controlled prospective studies with standardized acquisition, longitudinal coregistration, validated measurements, and clinical outcomes are required.

## Figures and Tables

**Table 1:** Baseline Characteristics of the Expanded Primary Imaging Cohort (*n* = 215).

Variable	Value
Age, y	71.1 ± 10.5
Male sex, *n* (%)	147 (68.4)
Baseline LDL-C level, mg/dL	117.4 ± 35.7
HbA1c level, %, mean ± SD	6.6 ± 1.3
HbA1c data available, *n*	153
Presence of diabetes mellitus, *n* (%)	64 (29.8)
Presence of hypertension, *n* (%)	123 (57.2)
Smoking history, *n* (%)	56 (26.0)
Prior PCI/CABG, *n* (%)	21 (9.8)
Presence of ACS, *n* (%)	144 (67.0)
Presence of CCS, *n* (%)	71 (33.0)
Statin use, *n* (%)	208 (96.7)
Ezetimibe use, *n* (%)	59 (27.4)
Prior evolocumab use, *n* (%)	6 (2.8)

LDL-C, low-density lipoprotein cholesterol; HbA1c, glycated hemoglobin; PCI, percutaneous coronary intervention; CABG, coronary artery bypass grafting; ACS, acute coronary syndrome; CCS, chronic coronary syndrome.

**Table 2:** Longitudinal Changes in LDL-C Levels and Plaque Volumes in the Expanded Primary Imaging Cohort.

Variable	Baseline, Mean (95% CI)	3 Months, Mean (95% CI)	15 Months, Mean (95% CI)	Overall *p*
LDL-C level, mg/dL	117.4 (113.3–121.6)	37.7 (33.5–41.9)	45.9 (39.5–52.3)	<0.0001
Total plaque volume, mm^3^	129.8 (112.4–147.3)	131.5 (114.0–148.9)	132.9 (114.7–151.1)	0.7127
NCP volume, mm^3^	73.5 (63.1–83.8)	74.5 (64.2–84.9)	65.8 (54.6–77.0)	0.0155
LDNC volume, mm^3^	27.3 (23.1–31.6)	27.2 (23.0–31.5)	22.7 (18.0–27.4)	0.0035
Calcified plaque volume, mm^3^	56.5 (45.6–67.3)	56.8 (46.0–67.6)	67.1 (55.9–78.2)	<0.0001

Values are expressed as model-estimated means with 95% CIs from random-intercept mixed-effects models. Overall *p*-values represent the categorical time effect. Respective observation counts at baseline, 3 months, and 15 months were 203, 193, and 77 for LDL-C and 186, 196, and 86 for the plaque metrics. Pairwise contrasts and adjusted *p*-values are provided in Table S2. LDL-C, low-density lipoprotein cholesterol; CI, confidence interval; NCP, non-calcified plaque; LDNC, low-density non-calcified plaque.

## Data Availability

The data presented in this study are available from the corresponding author upon reasonable request, subject to institutional and ethical restrictions.

## Supplementary Materials

The following supporting information can be downloaded at: https://www.mdpi.com/article/10.3390/diagnostics16183009/s1, Table S1: CCTA acquisition heart rate and radiation exposure; Table S2: Pairwise mixed-model contrasts in the expanded cohort; Table S3: Results of the tube voltage–adjusted mixed-effects sensitivity analysis; Table S4: Results of the correlation sensitivity analysis; Table S5: ACS-versus-CCS time-by-group interactions.

## Abbreviations

The following abbreviations are used in this manuscript:

PCSK9	Proprotein convertase subtilisin/kexin type 9
LDL-C	Low-density lipoprotein cholesterol
CCTA	Coronary computed tomography angiography
AI	Artificial intelligence
NCP	Non-calcified plaque
LDNC	Low-density non-calcified plaque
ACS	Acute coronary syndrome
CCS	Chronic coronary syndrome
HU	Hounsfield units
SE	Standard error
