# Supplementary material for: Serial Coronary Plaque Composition After Inclisiran Initiation Assessed by Automated CCTA: A Retrospective Pilot Study
Source: Diagnostics (Basel). 2026 Sep 17;16(18):3009. doi: 10.3390/diagnostics16183009 (PMC13604977; doi:10.3390/diagnostics16183009)
Supplement: Supplementary file 1 [file diagnostics-16-03009-s001.zip › diagnostics-4450941-supplementary.pdf]

Supplementary Statistical Results

Serial Coronary Plaque Composition After Inclisiran Initiation Assessed by Automated CCTA: A Retrospective Pilot Study

Table S1. CCTA acquisition heart rate and radiation exposure

Time point	CTDIvol (mGy)	DLP (mGy·cm)	Estimated effective dose (mSv)	Heart rate (bpm)
Baseline	21.6 ± 12.3 (n=88)	336.0 ± 199.0 (n=88)	4.70 ± 2.79 (n=88)	69.7 ± 16.4 (n=87)
3 months	20.2 ± 10.5 (n=98)	304.6 ± 171.2 (n=98)	4.26 ± 2.40 (n=98)	65.9 ± 12.4 (n=97)
15 months	16.7 ± 9.1 (n=87)	250.7 ± 136.5 (n=87)	3.51 ± 1.91 (n=87)	63.4 ± 11.0 (n=86)

Values are presented as mean ± standard deviation. The effective dose was estimated as DLP × 0.014 mSv/(mGy·cm). Data were retrospectively retrieved when available; therefore, denominators vary by time point and parameter. CTDIvol, volume CT dose index; DLP, dose-length product; bpm, beats/min.

Table S2: Pairwise mixed-model contrasts in the expanded cohort

Outcome	Contrast	Difference	95% CI	Unadjusted p	Bonferroni p
LDL-C (mg/dL)	Baseline vs. 3 months	-79.72	-84.61 to -74.83	4.479e-224	1.344e-223
LDL-C (mg/dL)	Baseline vs. 15 months	-71.52	-78.35 to -64.69	1.348e-93	4.045e-93
LDL-C (mg/dL)	3 vs. 15 months	8.20	1.34 to 15.06	0.01911	0.05734
Total plaque volume (mm^3)	Baseline vs. 3 months	1.63	-4.11 to 7.37	0.578	1
Total plaque volume (mm^3)	Baseline vs. 15 months	3.06	-4.69 to 10.82	0.4387	1
Total plaque volume (mm^3)	3 vs. 15 months	1.44	-6.22 to 9.10	0.7132	1
NCP volume (mm^3)	Baseline vs. 3 months	1.05	-3.49 to 5.58	0.6505	1
NCP volume (mm^3)	Baseline vs. 15 months	-7.67	-13.79 to -1.54	0.01414	0.04242
NCP volume (mm^3)	3 vs. 15 months	-8.72	-14.77 to -2.67	0.004744	0.01423
LDNC volume (mm^3)	Baseline vs. 3 months	-0.10	-2.24 to 2.05	0.9307	1
LDNC volume (mm^3)	Baseline vs. 15 months	-4.64	-7.54 to -1.74	0.001731	0.005194

LDNC volume (mm^3)	3 vs. 15 months	-4.54	-7.41 to -1.68	0.001893	0.005678
Calcified plaque volume (mm^3)	Baseline vs. 3 months	0.35	-2.61 to 3.31	0.8166	1
Calcified plaque volume (mm^3)	Baseline vs. 15 months	10.60	6.60 to 14.60	2.059e-07	6.178e-07
Calcified plaque volume (mm^3)	3 vs. 15 months	10.25	6.30 to 14.20	3.66e-07	1.098e-06

LDL-C, low-density lipoprotein cholesterol; vs., versus; NCP, non-calcified plaque; CI, confidence interval.

Table S3. Results of the tube voltage–adjusted mixed-effects sensitivity analysis

Outcome	Overall time p	Baseline-to-15-month difference	95% CI	Bonferroni p
Total plaque volume (mm^3)	0.4585	5.04	-3.34 to 13.42	0.7151
NCP volume (mm^3)	0.0900	-5.36	-12.32 to 1.60	0.3936
LDNC volume (mm^3)	0.0399	-4.02	-7.30 to -0.75	0.0480
Calcified plaque volume (mm^3)	<0.0001	10.27	6.16 to 14.38	<0.0001

The model included time as a categorical fixed effect, tube voltage (120 versus 140 kVp) as a time-varying fixed effect, and patient as a random intercept. The analysis included 107 patients and 278 observations (93 at baseline, 99 at 3 months, and 86 at 15 months). LDNC, low-density non-calcified plaque; NCP, non-calcified plaque; CI, confidence interval.

Table S4. Results of the correlation sensitivity analysis

Analysis	n	Coefficient	p
Pearson	64	r=0.259	0.0388
Spearman	64	rho=0.229	0.0687
Pearson excluding standardized-value outliers	56	r=0.147	0.2801
Spearman excluding standardized-value outliers	56	rho=0.144	0.2911

Prespecified standardized-value criterion: absolute z score >2 for either change score. Identified cases: 3, 8, 12, 14, 39, 46, 76, and 99.

Table S5. ACS-versus-CCS time-by-group interactions

Outcome	Wald chi-square (2 df)	p

LDL-C	14.198	0.0008
Total plaque volume	0.095	0.9538
NCP volume	0.581	0.7480
LDNC volume	1.646	0.4392
Calcified plaque volume	0.334	0.8463

LDNC, low-density non-calcified plaque; NCP, non-calcified plaque; LDL-C, low-density lipoprotein cholesterol.

Model diagnostics

Random-intercept models converged. Results of the residual Shapiro–Wilk tests were significant for all plaque metrics, indicating right-skew/non-normality. Accordingly, estimates were interpreted cautiously and compared with the complete-case nonparametric and prespecified sensitivity analyses. This pilot analysis does not establish equivalence, measurement repeatability, or a missing-at-random mechanism.
